# Supplementary material for: Direct interaction of a chaperone-bound type III secretion substrate with the export gate
Source: Nat Commun. 2022 Jun 2;13:2858. doi: 10.1038/s41467-022-30487-1 (PMC9163089; doi:10.1038/s41467-022-30487-1)
Supplement: Supplementary file 1 — Supplementary Information [file 41467_2022_30487_MOESM1_ESM.pdf]

Supplementary Information for

Direct interaction of a chaperone-bound type III secretion substrate with the export gate

Dominic Gilzer[1], Madeleine Schreiner[1], Hartmut H. Niemann[1]*

[1] Department of Chemistry, Bielefeld University, Universitaetstrasse 25, 33615 Bielefeld

* Corresponding author: hartmut.niemann@uni-bielefeld.de

Supplementary Tables

Supplementary Table 1. Data collection and refinement statistics. Values in parentheses are for the outer resolution shell.

	$YscX_{50}$:YscY (PDB: 7QIH)	$YscX_{32}$:YscY (PDB: 7QII)	$YscV_CX_{32}Y$ (PDB: 7QIJ)
**Diffraction images doi**	10.15785/SBGRID/905	10.15785/SBGRID/906	10.15785/SBGRID/907
**Data Collection**			
Beamline	BESSY BL14.2	SLS X06SA	DESY P13
Wavelength / Å	0.9184	1.0000	0.9762
Space Group	$P2_12_12$	$I4_122$	$P2_12_12_1$
Unit Cell			
a, *b*, *c* / Å	41.55, 140.83, 59.88	179.27, 179.27, 41.07	143.46, 324.92, 369.38
Resolution / Å	45.62-1.92 (2.03-1.92)	42.25-3.29 (3.49-3.29)	49.83-4.10 (4.21-4.10)
R_{meas}	0.102 (2.941)	0.130 (1.255)	0.113 (1.166)
$I/\sigma(I)$	16.40 (0.78)	14.55 (2.25)	11.15 (2.33)
$CC_{1/2}$	1.000 (0.446)	0.999 (0.773)	0.999 (0.791)
Completeness	0.997 (0.980)	0.998 (0.993)	0.996 (0.998)
Multiplicity	12.78 (11.29)	12.72 (13.04)	5.20 (5.50)
Wilson B-factor / Å²	39.51	111.39	163.79
**Refinement**			
No. reflections	26 281	5 357	135 236
No. reflections in free	1 384	535	6 767
R_{work}	0.2206	0.2296	0.3054
R_{free}	0.2624	0.2831	0.3251
No. atoms in AU			
Total	2 977	1 536	73 488
Protein	2 907	1 535	73 488
Water	70	1	0
Average B-factor / Å²			
Total	57.27	119.02	195.13
Protein	57.46	119.05	195.13
Water / Ions	49.37	65.33	-
r.m.s.d.			
Bond lengths / Å	0.002	0.012	0.002
Bond angles / °	0.39	1.77	0.60
Ramachandran			
Favored / %	97.69	95.12	90.90
Allowed / %	2.31	3.66	8.92
Outlier / %	0.00	1.22	0.18
Rotamer Outlier / %	0.32	0.65	2.63

Supplementary Table 2. Retention volume and apparent molecular masses calculated from analytical gel filtration. Retention volumes of peaks in size exclusion chromatograms (Fig. 6b) were attributed to proteins or protein complexes based on the apparent molecular mass calculated using a calibration run. Theoretical molecular masses of potential species are listed as well.

**Retention volume / mL**	**Apparent M_r / kDa**	**Assigned species**	**Theoretical M_r / kDa**
10 µM $YscV_C$			
14.93	57.7	$YscV_C$ dynamic	40.5 per monomer
10 µM $YscX_{32}Y$			
13.81	100.1	$(YscX_{32})_4(YscY)_4$	99.2
15.17	51.3	$(YscX_{32})_2(YscY)_2$	49.6
16.60	25.4	$(YscX_{32})_1(YscY)_1$	24.8
10 µM $YscV_C$ + 10 µM $YscX_{32}Y$			
9.88	692.5	$(YscV_C)_9(YscX_{32})_9(YscY)_9$	587.7
13.72	104.6	$YscV_CX_{32}Y$ dynamic	
14.93	57.7	$YscV_C$ dynamic	40.5 per monomer
		$(YscV_C)_1(YscX_{32})_1(YscY)_1$	65.3
16.43	27.6	$(YscX_{32})_1(YscY)_1$	24.8

Supplementary Table 3. Plasmids and constructs used in this study. All *Yersinia* genes were obtained by amplifying from the *Yersinia enterocolitica* pYVe227 virulence plasmid and sub-cloning using the denoted restriction sites. Mutations and deletions were generated via 'Round-the-horn (RTH) mutagenesis from previous plasmids. MBP = Maltose-binding protein; TEV = Tobacco etch virus protease cleavage site; fl = full-length.

Plasmid	Protein	Construct	Construct after TEV digest	Restriction sites used
pETM-40_*yscx*	$YscX_{fl}$	MBP-TEV-YscX	GAMG-YscX	*Nco*I, *Not*I
pETM-40_*yscx*32-122	$YscX_{32}$	MBP-TEV-$YscX_{32-122}$	GAMG-$YscX_{32-122}$	RTH
pETM-40_*yscx*50-122	$YscX_{50}$	MBP-TEV-$YscX_{50-122}$	GAMG-$YscX_{50-122}$	RTH
pETM-40_*yscx*32-110	$YscX_{32-110}$	MBP-TEV-$YscX_{32-110}$	GAMG-$YscX_{32-110}$	RTH
pETM-40_*yscx*50-110	$YscX_{50-110}$	MBP-TEV-$YscX_{50-110}$	GAMG-$YscX_{50-110}$	RTH
pACYCDuet-1_*yscy*	YscY	MG-His_6-G-$YscY_{2-114}$	-	*Nco*I, *Not*I
pACYCDuet-1_*yscy*_*yscv*356	YscY; $YscV_C$	MG-His_6-G-$YscY_{2-114}$ M-$YscV_{356-704}$	- -	*Nco*I, *Not*I, *Nde*I, *Xho*I
pACYCDuet-1_*yscy*_*yscv*356_R551A	YscY; $YscV_C^{R551A}$	MG-His_6-G-$YscY_{2-114}$ M-$YscV_{356-704}^{R551A}$	- -	RTH
pACYCDuet-1_*yscy*_*yscv*356_R551E	YscY; $YscV_C^{R551E}$	MG-His_6-G-$YscY_{2-114}$ M-$YscV_{356-704}^{R551E}$	- -	RTH
pETM-11_*yscv*356	$YscV_C$	MK-His_6-TEV-$YscV_{356-704}$	GAM-$YscV_{356-704}$	*Nco*I, *Not*I
pETM-11_*yscv*356-513	$YscV_{SD12}$	MK-His_6-TEV-$YscV_{356-513}$	GAM-$YscV_{356-513}$	RTH

Supplementary Figures

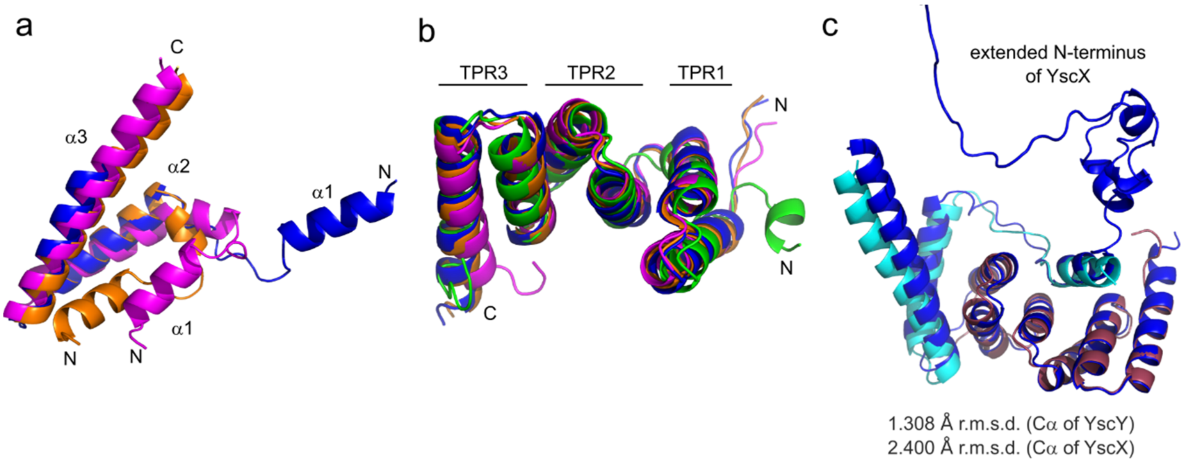

Supplementary Fig. 1. Models of YscX and YscY predicted by different programs. a Models of YscX predicted by AlphaFold 2 (blue), RoseTTAfold (magenta), and tFold (orange) align well for helices α2 and α3. The binding helix was predicted to either fold back or point away. Note that for RoseTTAfold and tFold, residues 50-122 were used, while AlphaFold was given the sequence only to residue 110. The model generated by I-TASSER does not align with the other three (not shown). **b** Models of YscY predicted by AlphaFold 2 (blue), RoseTTAfold (magenta), tFold (orange), and I-TASSER (green) all exhibit the same tandem-TPR fold. **c** Superimposed model of the deposited $YscX_{50}$:YscY structure (chain A and B, red and cyan) with the full-length AlphaFold 2 prediction of the complex. The models align well, especially for YscY (r.m.s.d. of 1.308 Å), with minor differences in α3 of YscX (r.m.s.d. of 2.400 Å). The N-terminus of YscX does not adopt a discernable fold in the predicted structure.

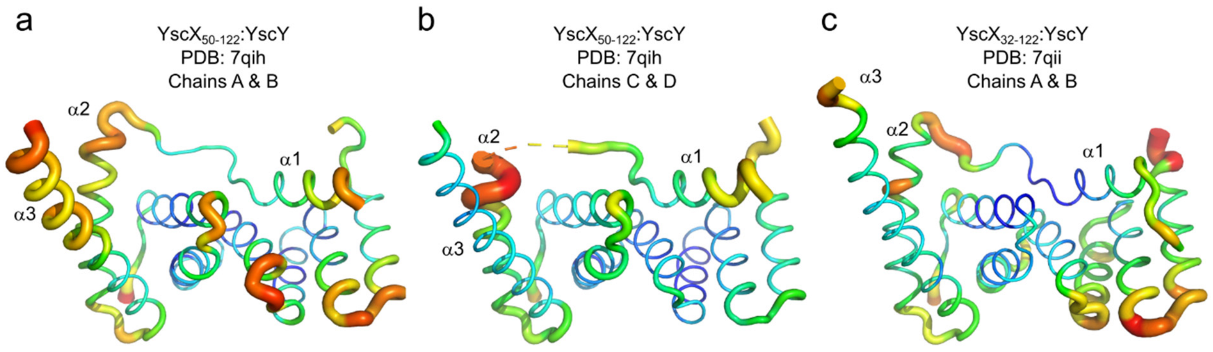

Supplementary Fig. 2. Comparison of B-factors in the two YscX:YscY structures. Blue color and thin tubes represent small B-factors, red and large tubes high B-factors. **a** $YscX_{50}$:YscY chain A and chain B. **b** $YscX_{50}$:YscY chain C and chain D. **c** $YscX_{32}$:YscY.

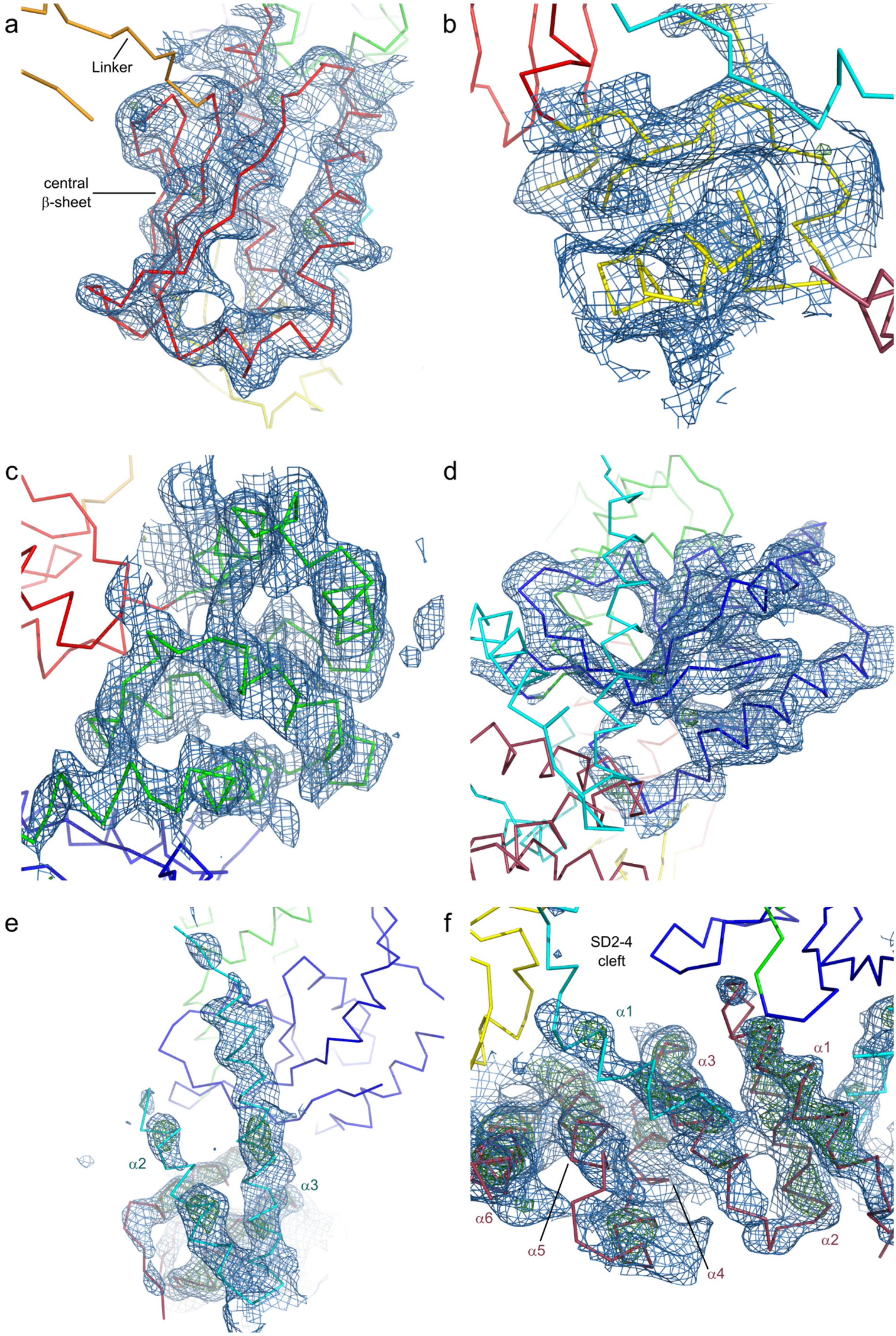

Supplementary Fig. 3. Electron density for the ternary YscV$_C$X$_{32}$Y complex. The 2mF_O-DF_C (1 σ, blue) and mF_O-DF_C (3 σ, green) shown were calculated after refinement with the placed YscV$_C$ model but

prior to placement of any of the 18 YscXY complexes. The final model is superimposed as ribbon. Density is presented around **(a)** SD1 (red), **(b)** SD2 (yellow), **(c)** SD3 (green), **(d)** SD4 (blue), and **(e+f)** the YscXY complex (cyan and dark red). The TPRs of YscY and the helices of YscX show clear density supporting their placement on the outside of the nonameric $YscV_C$ ring.

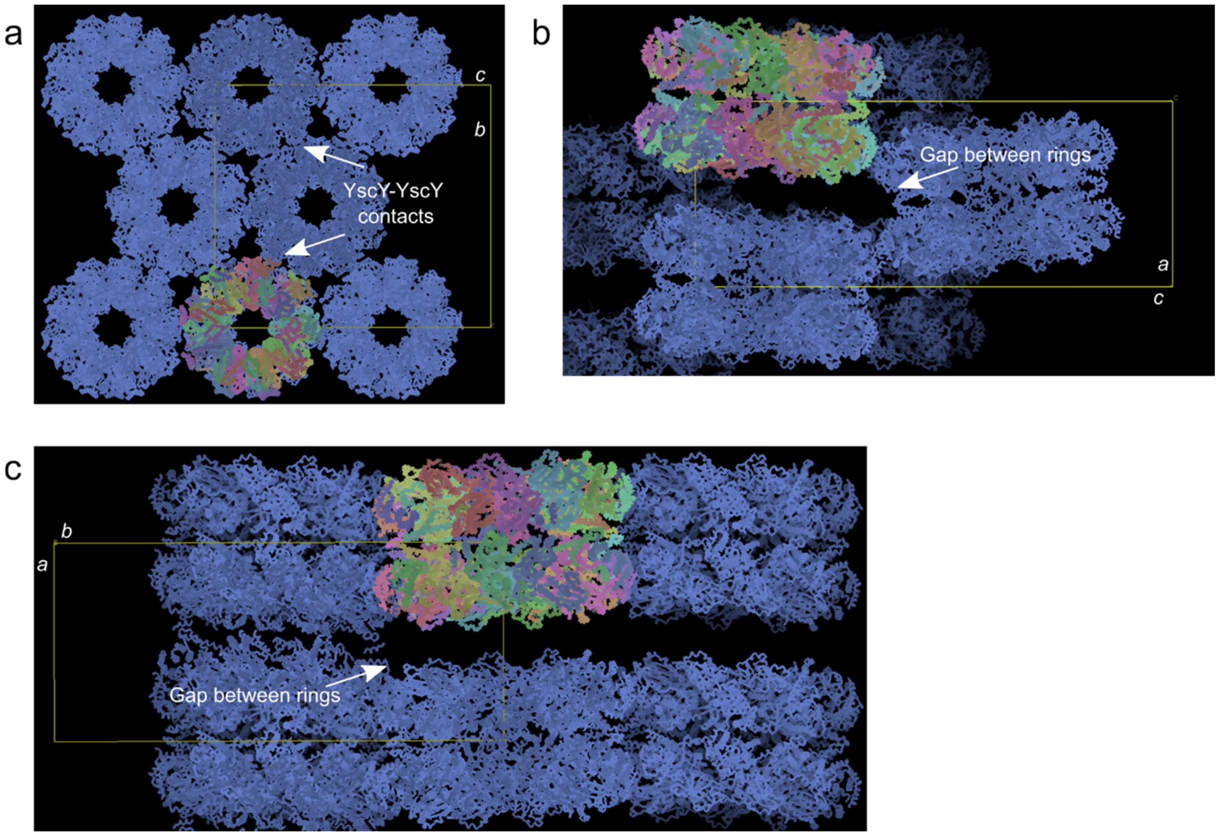

Supplementary Fig. 4. Packing of the YscV$_C$X$_{32}$Y crystal. Views along the **(a)** shortest, **(b)** middle and **(c)** longest axes of the unit cell are shown with the molecules portrayed as their Cα backbone. Almost all contact outside the double nonamer takes place on the outside of the ring with large solvent channels separating the membrane-proximal faces of adjacent rings.

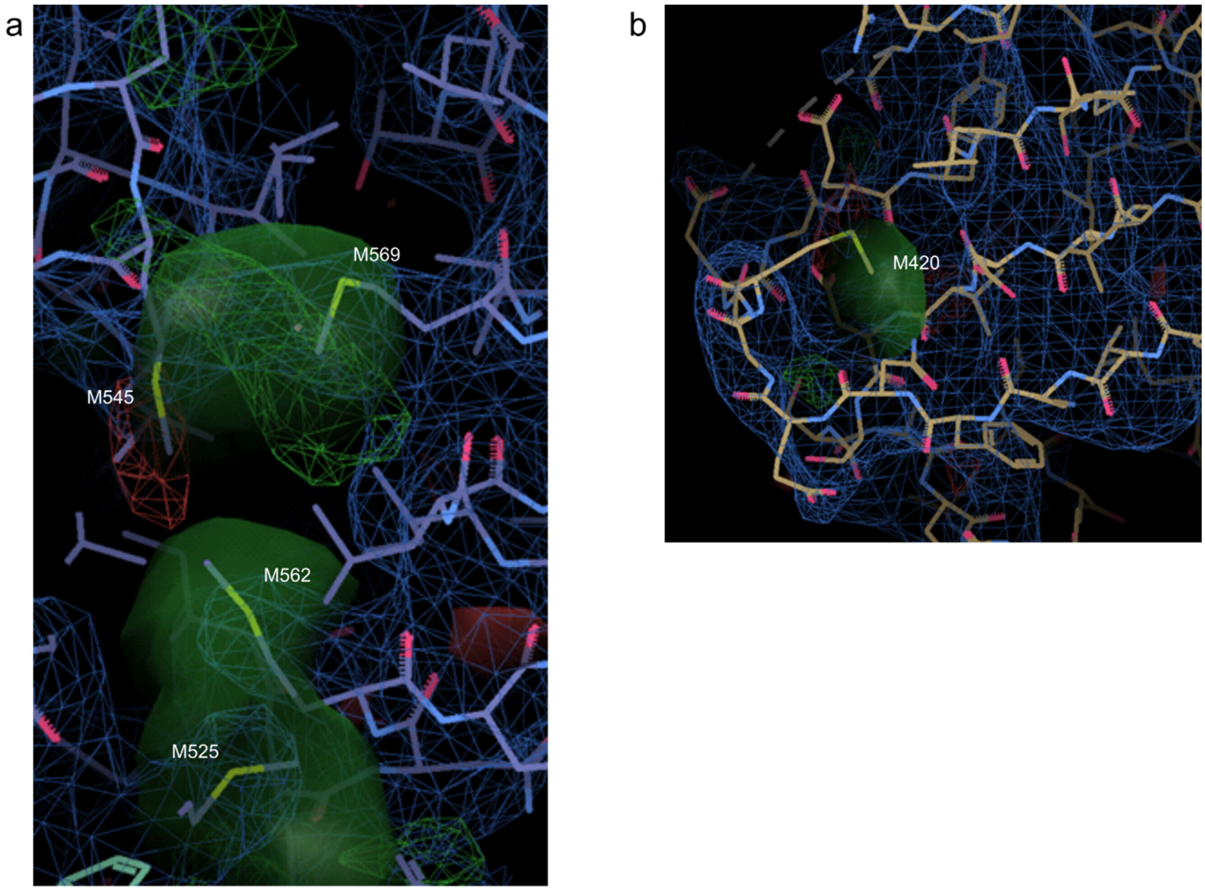

Supplementary Fig. 5. Anomalous difference density of a selenomethionine derivative of YscV$_C$X$_{32}$Y. The anomalous difference density (opaque green, 3 σ) of selenomethionine was employed to verify the sequence register of YscV$_C$ in the ternary complex structure. Due to a lack of non-start Met residues, this approach could not be employed for YscX and YscY. Exemplary density for **(a)** M525, M562, M545, and M569 (all SD3) and **(b)** M420 of SD1, which folds onto the central β-sheet.

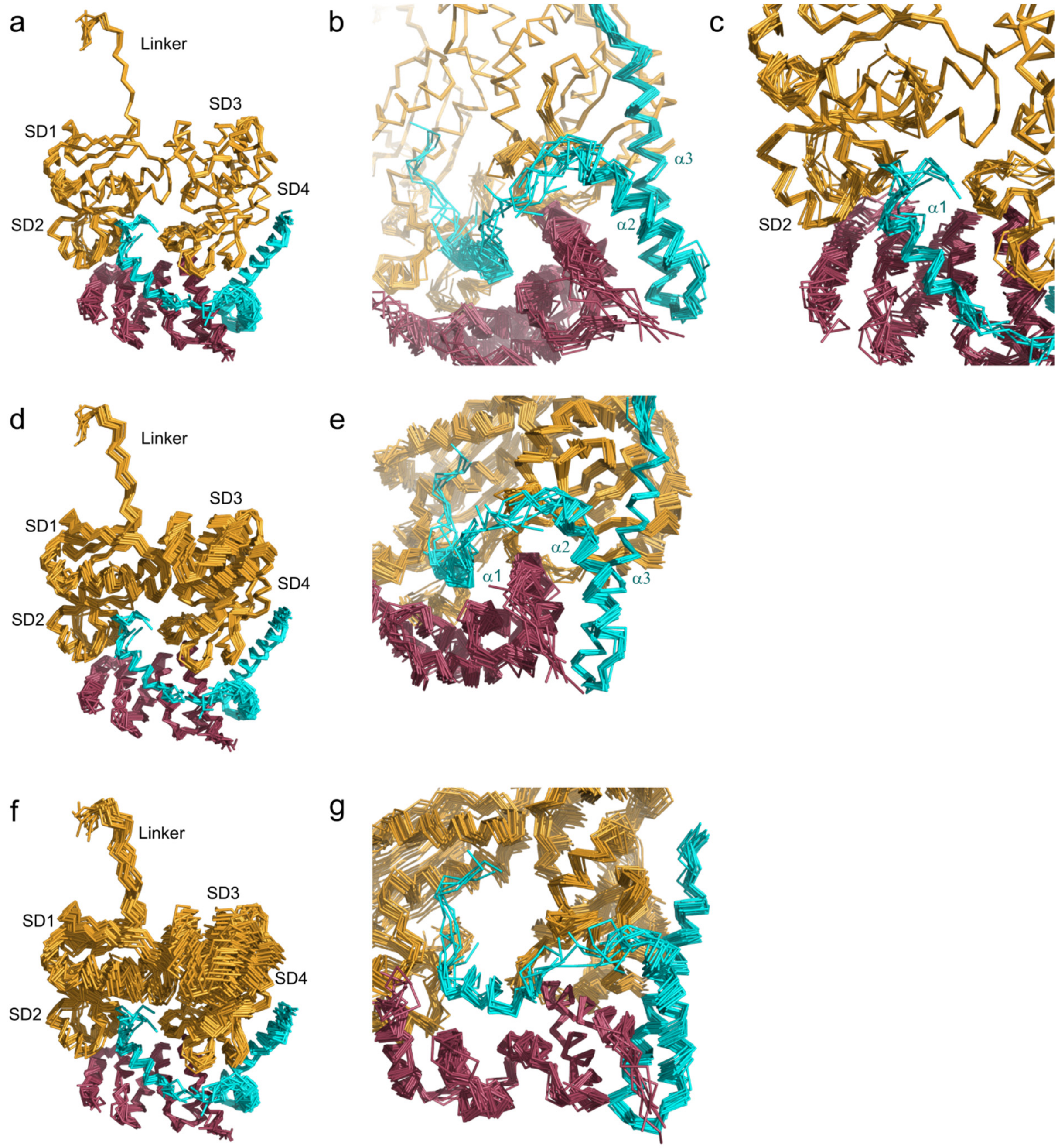

Supplementary Fig. 6. Superposition of chains in the $YscV_CX_{32}Y$ structure. All chains were aligned using **(a-c)** $YscV_C$, **(d-e)** $YscX_{32}$, or **(f-g)** His_6-YscY. For simplicity, the protein chains in the model were arranged so that every chain *x*A is a $YscV_C$ molecule, *x*B is $YscX_{32}$, and *x*C is YscY with *x* being a letter between A and R. Furthermore, chains *x*A, *x*B, and *x*C always produce a heterotrimeric complex. Chains A*y* to J*y* (*y* = A, B, or C for each protein species) constitute one nonameric ring, and chains K*y* to R*y* make up the second.

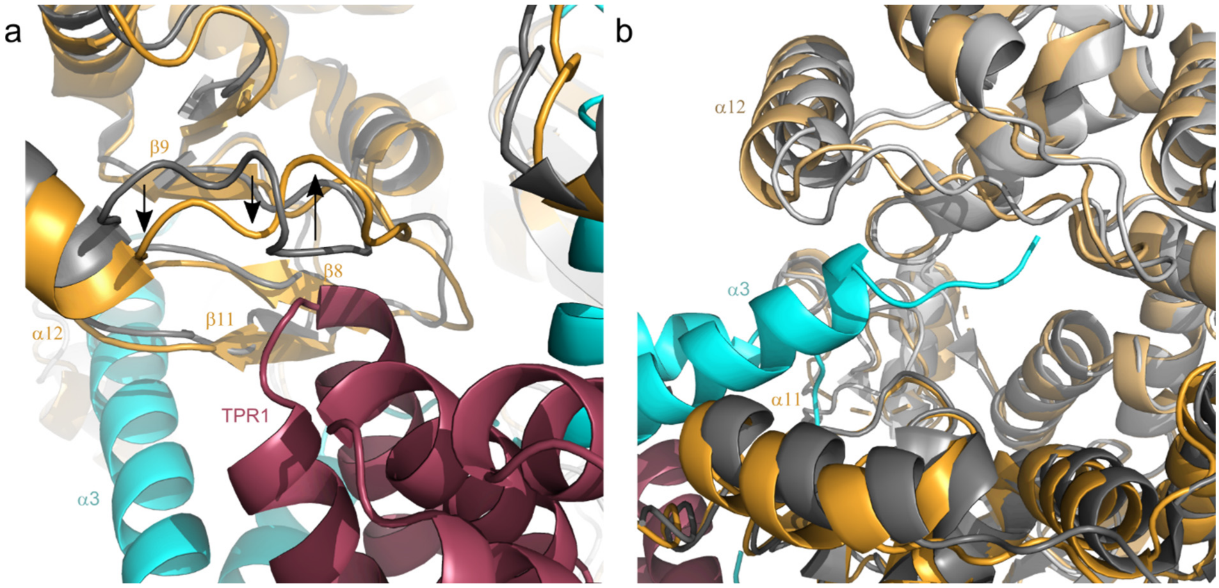

Supplementary Fig. 7. Minor movements within YscV$_C$ upon YscX:YscY binding. The structures of YscV$_C$ (grey shades, PDB: 7ALW) and YscV$_C$X$_{32}$Y (orange shades, cyan, and red) are superimposed with an r.m.s.d. of 1.4 Å using the nonameric ring. **a** Binding of YscY TPR1 to SD4 of YscV induces a shift of the loop between α12 and β9 away from YscY, possibly for steric reasons. **b** Binding of the α3 helix of YscX between YscV protomers induces only small rearrangements.

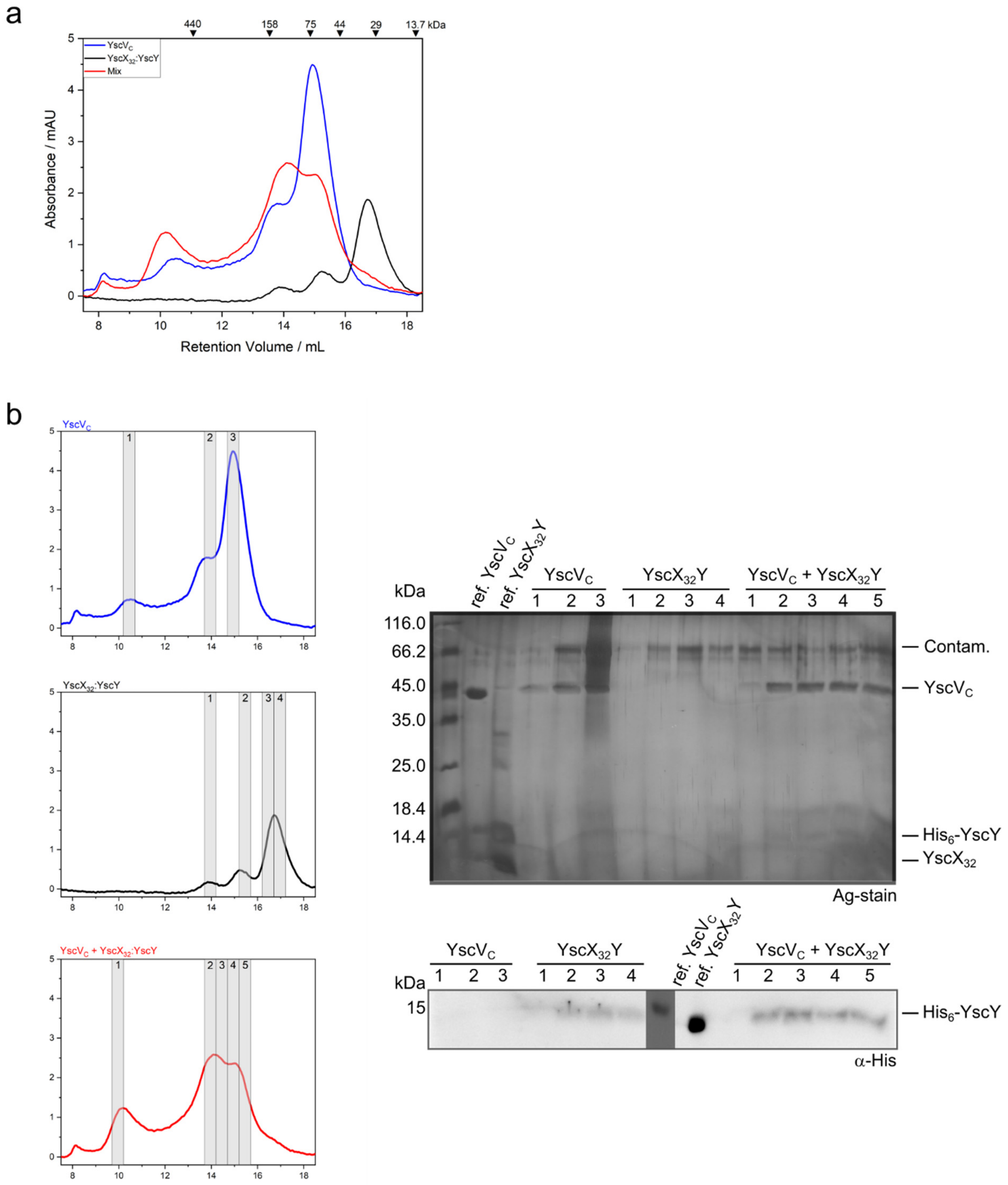

Supplementary Fig. 8. Analytical size exclusion chromatography of YscV$_C$, YscX$_{32}$Y, and all three proteins mixed. a A replicate of the three runs shown in Fig. 6b, where 10 µM YscV$_C$ (blue), YscX$_{32}$Y (black), or both (red) combined were loaded. **b** The three runs are shown separately with analyzed fractions highlighted as grey boxes. Silver-stained SDS-PAGE was used to identify contents of the individual peaks. . For comparison, the two proteins were also loaded as references. The contamination appearing as a band at roughly 66 kDa was introduced during the gel filtration runs, as it does not appear in the reference lanes of purified protein taken from the same aliquot as the gel filtration sample. Due to the low protein concentration in collected fractions, YscX$_{32}$ and YscY cannot be seen on the gel but the latter was detected via anti-His Western blot (lower panel) using mouse monoclonal anti polyHistidine-Peroxidase antibody A7058 (Sigma-Aldrich; RRID:AB_258326). The figure consists of

the lighter chemiluminescence image used to visualize the peroxidase-coupled antibody and the darker incident light (epi-white) image used to visualize the prestained marker bands in the middle. Source data are provided as a Source Data file. The results of the size exclusion chromatography were reproduced independently four times. An accompanying Western blot was carried out three times to validate the presence of His_6-YscY.
