# Supplementary material for: Direct interaction of a chaperone-bound type III secretion substrate with the export gate
Source: Nat Commun. 2022 Jun 2;13:2858. doi: 10.1038/s41467-022-30487-1 (PMC9163089; doi:10.1038/s41467-022-30487-1)
Supplement: Supplementary file 3 — Reporting Summary [file 41467_2022_30487_MOESM3_ESM.pdf]

nature portfolio

Corresponding author(s): Hartmut H. Niemann

Last updated by author(s): 21.04.2022

Reporting Summary

Nature Portfolio wishes to improve the reproducibility of the work that we publish. This form provides structure for consistency and transparency in reporting. For further information on Nature Portfolio policies, see our Editorial Policies and the Editorial Policy Checklist.

Statistics

For all statistical analyses, confirm that the following items are present in the figure legend, table legend, main text, or Methods section.

n/a	Confirmed	
☒	☐	The exact sample size (*n*) for each experimental group/condition, given as a discrete number and unit of measurement
☒	☐	A statement on whether measurements were taken from distinct samples or whether the same sample was measured repeatedly
☒	☐	The statistical test(s) used AND whether they are one- or two-sided<br>*Only common tests should be described solely by name; describe more complex techniques in the Methods section.*
☒	☐	A description of all covariates tested
☒	☐	A description of any assumptions or corrections, such as tests of normality and adjustment for multiple comparisons
☒	☐	A full description of the statistical parameters including central tendency (e.g. means) or other basic estimates (e.g. regression coefficient) AND variation (e.g. standard deviation) or associated estimates of uncertainty (e.g. confidence intervals)
☒	☐	For null hypothesis testing, the test statistic (e.g. *F*, *t*, *r*) with confidence intervals, effect sizes, degrees of freedom and *P* value noted<br>*Give P values as exact values whenever suitable.*
☒	☐	For Bayesian analysis, information on the choice of priors and Markov chain Monte Carlo settings
☒	☐	For hierarchical and complex designs, identification of the appropriate level for tests and full reporting of outcomes
☒	☐	Estimates of effect sizes (e.g. Cohen's *d*, Pearson's *r*), indicating how they were calculated

Our web collection on statistics for biologists contains articles on many of the points above.

Software and code

Policy information about availability of computer code

Data collection	X-ray crystallography: Reflections were measured at beamlines 14.2 (BESSY), X06SA (SLS), and P13 (DESY) using the local installation of MxCube (Gabadinho et al., 2010).<br><br>Size exclusion chromatography: Runs were carried out using ÄKTA systems with co-distributed software UNICORN 5.20 (Cytiva).
Data analysis	X-ray crystallography:<br>Data were processed using XDSAPP version Mar 15, 2019 (Krug et al., 2012) for YscX50:YscY or XDS version Feb 5, 2021 and version Jan 26, 2018 (Kabsch, 2010) for YscX32:YscY and YscVXY, respectively.<br>Phasing was done in Phaser 2.8.3 (McCoy et al., 2007) within the Phenix 1.19.2-4158 environment (Liebschner et al., 2019) using a locally generated AlphaFold v2.0 (Jumper et al., 2021) model.<br>Buccanneer v1.5 (Cowtan, 2006) in the CCP4i GUI 7.1.0.16 (Winn et al., 2011) was employed for automated model building.<br>Models were refined in phenix.refine 1.19.2_4158 (Adams et al., 2010) and built manually in WinCOOT 0.9.6 (Emsley et al., 2010).<br>Resolution cutoff was determined with PAIREF 1.3.7 (Malý et al., 2020, Malý et al. 2021).<br>Images were generated in PyMOL 2.5.0 (Schrödinger LLC) with OpenGL 4.5 and GLSL 4.50.<br><br>Alignments were calculated using ClustalW 0(1.2.4) (SIevers et al. 2011) and visualized with ESPript 3.0 (espript.ibcp.fr) (Robert et al. 2014).<br><br>Size exclusion chromatography: Plots were generated using Origin 2020b (OriginLab).

For manuscripts utilizing custom algorithms or software that are central to the research but not yet described in published literature, software must be made available to editors and reviewers. We strongly encourage code deposition in a community repository (e.g. GitHub). See the Nature Portfolio guidelines for submitting code & software for further information.

Data

Policy information about availability of data

All manuscripts must include a data availability statement. This statement should provide the following information, where applicable:

- Accession codes, unique identifiers, or web links for publicly available datasets
- A description of any restrictions on data availability
- For clinical datasets or third party data, please ensure that the statement adheres to our policy

The coordinates and structure factors of all structures have been deposited in the Protein Data Bank (pdb.org) under the accession codes 7QIH [http://doi.org/10.2210/pdb7QIH/pdb] for YscX50:YscY, 7QII ([http://doi.org/10.2210/pdb7QII/pdb] for YscX32:YscY, and 7QIJ [http://doi.org/10.2210/pdb7QIJ/pdb] for YscVXY, and will be released upon acceptance of the manuscript.

Diffraction images are available from the SB Grid Data Bank (data.sbgrid.org) with Data ID 905 [https://doi.org/10.15785/SBGRID/905] for YscX50:YscY (7QIH), Data ID 906 [https://doi.org/10.15785/SBGRID/906] for YscX32:YscY (7QII), and Data ID 907 [https://doi.org/10.15785/SBGRID/907] for YscVXY. The images will be released upon acceptance of the manuscript.

To solve the phase problem of the ternary complex, the structure of nonameric YscVc with PDB ID 7ALW was used. This is publicly accessible in the PDB [http://doi.org/10.2210/pdb7ALW/pdb].

The structures used to generate Fig. 8 and Fig. 9 are available from the wwPDB.
Fig. 8a: 2P58 [http://doi.org/10.2210/pdb2P58/pdb] (YscEFG)
Fig. 8b: 3WXX [http://doi.org/10.2210/pdb3WXX/pdb] (AcrH:AopB)
Fig.9a: 7ALW [http://doi.org/10.2210/pdb7ALW/pdb] (YscVC nonamer)
Fig. 9b: 6CH2 [http://doi.org/10.2210/pdb6CH2/pdb] (FlhA:FliT:FliD)
Fig. 9c: 6CH3 [http://doi.org/10.2210/pdb6CH3/pdb] (FlhA:FliS:FliC)
Fig 9d: 6WA9 [http://doi.org/10.2210/pdb6WA9/pdb] (CdsV:CdsO)

Source data (uncropped gels and blots) are provided as Source Data file.

Field-specific reporting

Please select the one below that is the best fit for your research. If you are not sure, read the appropriate sections before making your selection.

☒ Life sciences ☐ Behavioural & social sciences ☐ Ecological, evolutionary & environmental sciences

For a reference copy of the document with all sections, see nature.com/documents/nr-reporting-summary-flat.pdf

Life sciences study design

All studies must disclose on these points even when the disclosure is negative.

Sample size

Diffraction data for all structures was collected from a single crystal each to ensure complete data and high redundancy.

Sample size estimation by statistical methods was not relevant for this work, as it does not use statistical evaluation of effects between two or more groups. All biochemical experiments were repeated to verify the results (see Replication). The number of independent experiments are specified in the figure legends.

Data exclusions

The YscVXY dataset was truncated to remove the last 800 of the collected frames due to radiation damage.

Reflections were automatically discarded using the internal standards for outlier detection of the programs used.

Resolution cutoff was based on CC1/2 statistics and <(I)/(sigma I)> for the YscX50:YscY and YscX32:YscY structure. For the YscVXY structure, the resolution cutoff was determined using paired refinements as implemented in the software PAIREF.

Replication

Crystallization:
- YscX50:YscY: Purification was repeated several times, but crystallization results were inconsistent between batches. Some batches required seeding from previously obtained crystals or did not produce crystals at all. However, crystals harvested from different batches were of similar diffraction quality. We could not determine the reason behind differences in crystal growth.
- YscX32:YscY: Purification was repeated several times, but reproduction of this crystal form could not be achieved.
- YscVXY: Crystals could be reproduced in a variety of conditions, but only very few crystals diffracted to a useable resolution.

Data collection:
We tested about 20 crystals of YscX50:YscY, 10 crystals of YscX32:YscY and about 100 crystals of YscVXY.
Crystallographic data collection was carried out to ensure high completeness and multiplicity of the data. Please refer to Supplementary Table 1.

Biochemical experiments:
Size exclusion chromatography of the mixture of YscVSD12 and YscX32:YscY (Fig. 6b lower graph) was performed twice with consistent results.
All other biochemical experiments (pull down assays; other size exclusion chromatography runs; Western blot) were independently

performed three or four times with consistent results. The number of independent experiments is noted in the legends of Fig. 6, Fig. 7 and Supplementary Fig. 8.

Randomization	This is not relevant to our study, because samples were not allocated into experimental groups.
Blinding	This is not relevant to our study, because there was no group allocation.

Reporting for specific materials, systems and methods

We require information from authors about some types of materials, experimental systems and methods used in many studies. Here, indicate whether each material, system or method listed is relevant to your study. If you are not sure if a list item applies to your research, read the appropriate section before selecting a response.

Materials & experimental systems

n/a	Involved in the study
☐	☒ Antibodies
☒	☐ Eukaryotic cell lines
☒	☐ Palaeontology and archaeology
☒	☐ Animals and other organisms
☒	☐ Human research participants
☒	☐ Clinical data
☒	☐ Dual use research of concern

Methods

n/a	Involved in the study
☒	☐ ChIP-seq
☒	☐ Flow cytometry
☒	☐ MRI-based neuroimaging

Antibodies

Antibodies used

Anti polyHistidine-Peroxidase A7058 (mouse IgG2a) from Sigma-Aldrich (RRID:AB_258326; http://antibodyregistry.org/AB_258326)

Validation

The Product Specification notes: "Purchaser must determine the suitability of the product for its particular use.". The Certificate of Analysis for the batch used for the blot in Supplementary Fig. 9 (batch 080M4836) notes that in the QC Test on 25-Aug-2010 the Results match the Specification, i.e.
"- Immunoblot direct:
- bacterial preparation: E.Coli expr.polyhistidine fusion protein
- substrate: AEC
- working dilution 1/4000.0
- band appearance: one band
- Recommended Retest Date: 2/2012".
We last verified the antibody's specificity on 11-Dec-2019 using whole-cell lysate of E. coli expressing a His6-tagged version of SctY from Photorhabdus luminescens. The blot showed a single band at the expected height thus matching the Specification.
For the experiment reported in this paper, we included as positive control purified His6-tagged YscY in our blot (Supplementary figures 8+9, lane "ref-YscX32Y").
